# From Defeat to Empowerment: User Experiences From a Health Promotion Intervention for People With Dementia

**DOI:** 10.1093/geroni/igaa057.529

**Published:** 2020-12-16

**Authors:** Martine Kajander, Martha Therese Gjestsen, Ingelin Testad

**Affiliations:** 1 Centre for Age-related Medicine - SESAM, Stavanger University Hospital, Stavanger, Norway, Sandnes, Norway; 2 Centre for Age-related Medicine - SESAM, Stavanger University Hospital, Stavanger, Norway, Stavanger, Norway; 3 Centre for Age-Related Medicine - SESAM, Stavanger University Hospital, Stavanger, Norway

## Abstract

Empowering people with early-stage dementia through the provision of information and support has gained an increasing focus as the number of people with dementia increases worldwide. Health Promotion is a mean to empower the person affected to take an active role in the situation, and taking steps themselves to adjust and cope with the condition. The aim of this study was to explore the experiences of people with early-stage dementia provided with support and information through a 12-week Health Promotion course. Data comprises separate individual semi-structured interviews with 32 people with dementia after attending the course. For each participant, a carer was also interviewed. Interviews were analysed using systematic text condensation. Four categories emerged from the analysis. These were: (I) bridging the post- diagnostic information gap, (II) promoting healthy behaviours, (III) meeting others with early-stage dementia, and (IV) coming to terms with the diagnosis. The results demonstrated that the intervention was well received by participants; learning about dementia, meeting others in the same situation and focussing on maintaining a healthy lifestyle empowered and motivated participants. The participants’ carers found the course booklet especially useful and it improved family communication. In conclusion, a 12-week Health Promotion course has the potential to empower people with dementia to cope with their condition through the provision of information, peer-support, which in turn can improve family communication and ease the process of accepting the diagnosis.

